# Association Between Facial Biotype and Condylar Spaces in Orthodontic Patients: A Cross-Sectional Cone-Beam Computed Tomography (CBCT) Study

**DOI:** 10.7759/cureus.98388

**Published:** 2025-12-03

**Authors:** Patricia Lizbeth Aguilera-González, Luis Pablo Cruz-Hervert, Silvia Paulina Martínez-Contreras, Gerardo Martínez-Suárez, Carla Monserrat Ramírez-Martínez, Luis Fernando Jacinto-Alemán, Beatriz Raquel Yáñez-Ocampo, Juan Carlos Solorio-Quezada, Aidé Karina Valdez-Sánchez, Ronald R Ramos Montiel, María Eugenia Jiménez-Corona

**Affiliations:** 1 Orthodontics and Maxillofacial Orthopedics, Universidad Cuauhtémoc San Luis Potosí, San Luis Potosí, MEX; 2 Division of Postgraduate Studies and Research, Faculty of Dentistry, Universidad Nacional Autónoma de México, Mexico City, MEX; 3 Department of Epidemiology, Instituto Nacional de Cardiología Ignacio Chávez, Mexico City, MEX; 4 Faculty of Higher Studies Iztacala, Universidad Nacional Autónoma de México, Mexico City, MEX; 5 School of Dentistry, Universidad Anáhuac Veracruz, Xalapa, MEX; 6 Orthodontics, Universidad Católica de Cuenca, Cuenca, ECU

**Keywords:** bilateral symmetry, cbct, cluster analysis, condylar position, condylar spaces, facial biotype, orthodontic

## Abstract

Background: Condylar position is a key element in diagnosis and treatment planning in orthodontics and rehabilitation. Facial biotype influences features such as muscular strength and vertical dimension. Evidence associated with a possible relationship between them is still limited, which keeps the topic clinically and scientifically relevant.

Objective: This was an observational, analytical, cross-sectional study conducted using CBCT records of adult patients treated at the Orthodontics and Maxillofacial Orthopedics Graduate Clinic of Universidad Cuauhtémoc, San Luis Potosí, Mexico. It aimed to evaluate the association between facial biotype and condylar position in orthodontic patients using cone-beam computed tomography (CBCT) and to identify condylar space distribution patterns, bilateral symmetry, and factors associated with ideal condylar position and specific pattern membership.

Materials and methods: CBCT scans of 100 adult orthodontic patients were analyzed in this study. The anterior (AS), superior (SS), and posterior (PS) condylar spaces were measured on both sides. Facial biotypes were determined using Ricketts cephalometric analysis. Descriptive statistics, analysis of variance (ANOVA), cluster analysis, bilateral symmetry assessment, and multivariate regression models were used in the study.

Results: Dolichofacial patients exhibited a significantly smaller right AS than those with the brachyfacial and mesofacial types (p = 0.011). Cluster analysis revealed three condylar space patterns: Group 1 (close to ideal), Group 2 (moderately enlarged), and Group 3 (enlarged) patterns. Bilateral concordance was observed in 37 (37%) patients in G1, 20 (20%) in G2, and five (5%) in G3. Dolichofacial biotype significantly increased the likelihood of bilateral G1 membership (odds ratio (OR) = 4.08, p = 0.041). None of these factors consistently predicted strict compliance with Ikeda's ideal values.

Conclusions: Facial biotypes selectively influence condylar position, with dolichofacial patients showing reduced AS. The condylar position exhibits distinct subgroup patterns, and bilateral symmetry is not always present. The dolichofacial biotype was the main predictor of condylar configuration. These findings emphasize the need for individualized joint evaluation, interpretation of the biotype as a modulator of the condylar-fossa relationship, and reliance on distribution patterns rather than rigid values for orthodontic diagnosis and treatment planning.

## Introduction

The position of the mandibular condyle within the glenoid fossa is fundamental to understanding the functional dynamics of the temporomandibular joint (TMJ). In orthodontics, this aspect is particularly relevant, as it influences treatment stability and the planning of dental and mandibular movements, and in some cases, it is related to the appearance of signs and symptoms of TMJ dysfunction. Different methods have been proposed to characterize this position, among which measurement of the anterior, superior, and posterior joint spaces has proven to be a valuable tool. These quantitative parameters make it possible to precisely describe the condylar location, establish inter-individual comparisons, and analyze adaptive changes after orthodontic therapy [1].

New methods have been proposed for evaluating these dimensions using cone-beam computed tomography (CBCT). Unlike conventional radiography or medical CT, CBCT provides high-resolution three-dimensional images with a relatively low radiation dose, enabling reliable and reproducible analyses of condylar structures [2]. CBCT allows for the direct measurement of joint spaces, exploration of bilateral symmetry, and evaluation of the influence of anatomical or functional variables [3]. Therefore, CBCT has become the tool of choice for research aimed at establishing clinically relevant parameters in orthodontics and dentofacial orthopedics [2,4,5], particularly for skeletal or volumetric assessment [6].

Ikeda et al. [1] established reference values for condylar spaces in young, asymptomatic subjects considered representative of an “ideal position”: 1.3 mm for the anterior space, 2.5 mm for the superior space, and 2.1 mm for the posterior space. These values have been widely used as a standard for assessing the condylar location and have served as a reference framework in diagnostic and therapeutic studies. However, questions arise regarding their applicability in orthodontic patients, who often present with malocclusions, varied skeletal patterns, and, in some cases, articular adaptations related to age or accumulated functional loads [1,7,8].

Previous studies have reported significant changes in condylar position after orthodontic therapy. In particular, in patients with Class II Division 2 malocclusion, a retropositioned condyle is frequently observed before treatment, characterized by an increase in the posterior space and a reduction in the anterior space. After treatment, there is a tendency toward condylar recentralization, with a decrease in the posterior space, an increase in the anterior space, and a pattern of adaptive reconstruction involving increased condylar volume and articular surface [9,10]. These findings demonstrate that the condylar position is adaptive and dynamic, responds to therapeutic interventions, and reflects bone remodeling processes.

Other studies conducted in patients with craniofacial asymmetries and conditions such as craniofacial microsomia or mandibular hyperplasia have shown divergent condylar growth trajectories compared with those in symmetric individuals. It has been reported that the hyperplastic side may exhibit up to 20% greater condylar growth than the contralateral side, whereas in hemifacial microsomia, the affected side shows significantly reduced growth of the posterior ramus [11]. These data reinforce the idea that the condylar position cannot be considered uniform but is modulated by morphological, functional, and pathological factors.

Head posture has also been identified as a modulator of the condylar position. Ohmure et al. [12] demonstrated that a forward head posture induced posterior displacement of the condyle compared with the position obtained in a natural posture, highlighting the influence of functional determinants on the condylar relationships. These findings, together with reports on the variability associated with skeletal patterns, underscore the need to consider individual factors in condylar position assessment.

Despite the available evidence, there are important gaps in the literature. It is crucial to determine whether facial biotypes systematically modify the condylar spaces. Although biotypes are known to influence mandibular morphology and articular eminence inclination [13,14], evidence regarding their relationship with condylar position remains contradictory. Some authors have reported that patients with dolichofacial biotypes present with a smaller anterior space, whereas others have found no significant differences between the biotypes [15,16]. This gap limits the clinical interpretation of the condylar measurements based on individual facial patterns.

In addition, to the best of our knowledge, no study has investigated whether bilateral specific patterns of condylar space distribution exist. To date, most studies have reported mean values or ranges; however, few have explored the possibility that specific combinations of the three spaces may form distinguishable articular phenotypes in the hip joint. Therefore, it is important to identify whether clusters exist that allow patients to be classified into clinically meaningful categories, rather than relying solely on comparisons with ideal values derived from small and specific populations.

There is limited evidence regarding condylar morphology and position in orthodontic patients. Although condylar asymmetry has been described in craniofacial deformities and pathological conditions [11,17], its bilateral concordance in orthodontic patients has not been systematically evaluated yet. Likewise, while the parameters proposed by Ikeda et al. [1] are widely cited as reference standards, studies suggest that only a minority of patients meet these values [7,8], and their applicability to the general population remains uncertain. Furthermore, no study has assessed the factors associated with the ideal condylar position, such as age, sex, skeletal class, or head posture [10,12], and multivariate models integrating these variables are still lacking. Together, these gaps highlight the need for broader and more comprehensive investigations to clarify the clinical relevance of condylar position. Therefore, it is important to generate evidence that not only expands knowledge but also has practical applications. In orthodontics, precise characterization of the condylar position makes it possible to anticipate articular variations, adjust treatment mechanics, and establish criteria for long-term stability. CBCT provides reliable three-dimensional information for joint-space evaluation, and its accuracy and reproducibility for linear measurements have been demonstrated in other studies [3,6].

In this context, the primary aim of our study was to evaluate the association between facial biotype and the condylar joint spaces, specifically the anterior (AS), superior (SS), and posterior (PS) spaces, in orthodontic patients using the Ikeda method applied to CBCT images. We hypothesized that there would be no significant association between facial biotype and the condylar joint spaces. The overall objective of this study was to analyze whether facial biotypes are related to differences in anterior, superior, and posterior condylar spaces. In addition, the following specific objectives were proposed: to identify the existence of condylar space distribution patterns through cluster analysis, to determine whether the identified patterns were symmetrically reproduced in both TMJs, to estimate the proportion of patients who met Ikeda’s ideal values, and to build multivariate models that identified the factors associated with both ideal condylar position and membership in a specific pattern. If such differences are confirmed, treatment biomechanics may need to be adjusted by integrating facial biotype and condylar position into diagnosis and planning.

## Materials and methods

Study design 

This was an observational, analytical, cross-sectional study conducted using CBCT records of adult patients treated at the Orthodontics and Maxillofacial Orthopedics Graduate Clinic of Universidad Cuauhtémoc, San Luis Potosí, Mexico. This design was chosen for its suitability in exploring morphological associations at a single time point, following methodologies described in previous studies of the temporomandibular joint. The protocol was approved by the University’s Ethics Committee (CEI-UCSLP-2024) and was conducted in accordance with the Declaration of Helsinki.

Population and sample selection

The study population consisted of 422 CBCT scans obtained between 2021 and 2023. Simple random sampling was used to minimize selection bias. The inclusion criteria were as follows: age between 18 and 50 years, complete permanent dentition, no history of orthodontic treatment, and no signs and symptoms of temporomandibular disorders. The exclusion criteria were the presence of craniofacial syndromes, malformations, or pathologies affecting mandibular growth; dental agenesis (including supernumerary teeth, microdontia, or macrodontia); history of orthognathic surgery or facial trauma; and tomographic findings preventing adequate evaluation of joint structures.

Sample size calculation

The sample size was estimated for a multiple linear regression model with condylar joint space (mm) as the dependent variable and facial biotype as the main independent variable, adjusting for potential confounders. AS, SS, and PS spaces were considered co-primary outcomes and were assumed to have similar variability. This assumption was based on the reference values reported by Katsumi et al. (2009) in asymptomatic adults, where PS was 2.1 mm (±0.3), SS 2.5 mm (±0.5), and AS 1.3 mm (±0.2). Because these estimates derive from a small, highly selected sample (n = 25), they were used to inform the expected scale and dispersion of the measurements rather than to fix a precise target difference. Therefore, we adopted a conventional medium anticipated effect size for multiple regression (f² ≈ 0.15), which corresponds to an adjusted R² of approximately 0.13 for a model including six predictors (facial biotype plus age, sex, skeletal class, and side, with the possibility of one additional covariate entering the saturated model if p < 0.20). Using α = 0.05 and 80% power, the minimum required sample size was 97 subjects, calculated using an online calculator for a priori sample size estimation for multiple regression (Version 4.0). We included 100 participants to ensure adequate power in case of missing or unusable data [18].

CBCT data acquisition

Images were obtained using a NewTom VGi scanner (Verona, Italy) under the following standardized protocol: 110 kV, pulsed mode 1-20 mA, scan time of 18-26 seconds, and a field of view of 15 × 15 cm. The CBCT scans were acquired with a voxel size (effective slice thickness) of 0.30 mm x 0.30 mm. The patients were positioned in maximum habitual intercuspation, with the head in a natural posture and the Frankfurt plane parallel to the floor, using chin support to avoid movement. The images were stored in the DICOM format and analyzed using 3D Slicer software (version 5.6.1, macOS).

Measurement procedure of articular spaces

**Figure 1:**
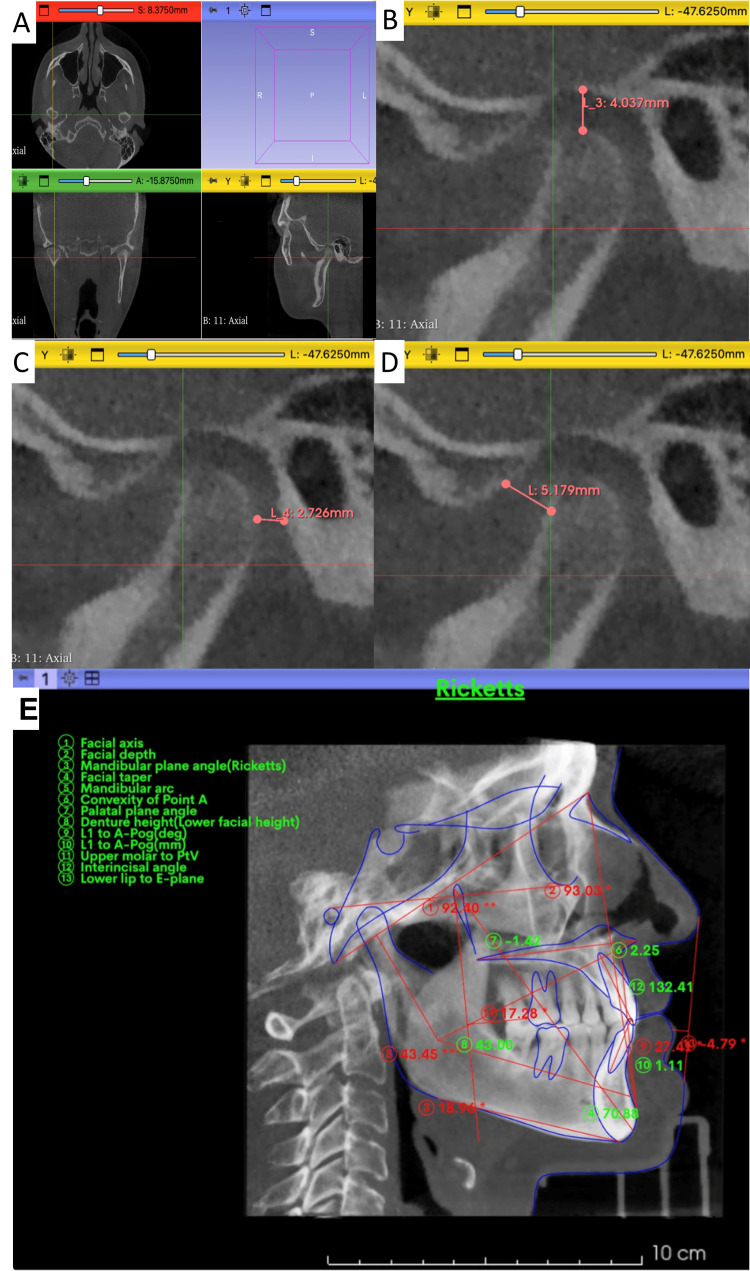
Graphic description of the process for recording condylar measurements and cephalometric tracing with AI using WebCeph. Footnote: Panels A–D: Sequential steps performed in 3D Slicer to assess condylar spaces. Panel A shows the identification of reference planes and image centering prior to measurement. Panel B illustrates the placement of landmarks and creation of linear measurements for anterior, superior, and posterior joint spaces. Panel C presents the finalized measurements and their spatial relationships within the sagittal and coronal views. Panel D displays the last condylar measurement obtained. Panel E: Cephalometric tracing performed in WebCeph™ (Assemble Circle Co., Ltd., Gyeonggi-do, Republic of Korea) after prior image calibration. The illustrated analysis corresponds to the Ricketts analysis. Point identification and both angular and linear measurements were automatically generated by an artificial intelligence algorithm integrated into the software. Image credits: Dr. Patricia Lizbeth Aguilera-González, and Dr. Luis Pablo Cruz-Hervert

The 3D Frankfurt plane (Po-Or) was used as a reference. In 3D Slicer (version 5.7), sagittal slices passing through the condylar center were selected. The condylar center was identified using a standardized protocol: first locating the widest mediolateral diameter of the condylar head, then determining the midpoint between the medial and lateral poles on the axial slice, and finally projecting this midpoint onto the sagittal plane for spatial consistency. Using the line tool from the Markups module, three anatomical points were marked on the condylar surface (anterior, superior, and posterior), each of which was connected to the corresponding wall of the glenoid fossa to obtain linear measurements of the anterior (AS), superior (SS), and posterior (PS) joint spaces. All measurements were performed bilaterally, following the technique described by Ikeda and Kawamura [7]. Additionally, the Horizontal Condylar Axis (HCA) is defined as the angle between the long axis of the condyle, determined by its maximum mediolateral length on axial CBCT, and the coronal plane, drawn perpendicular to the midsagittal plane, as defined by Westesson et al. [19] and Kristensen et al. [20], as shown in Figure 1.

After identifying the clusters based on condylar spaces through prior exploratory factor and cluster analyses, we defined a symmetrical condylar space distribution when both the left and right condylar spaces were assigned to the same cluster group. If this condition was not satisfied, the distribution was considered asymmetrical.

Cephalometric variables and facial biotype classification

Facial biotypes were determined using the Ricketts cephalometric analysis in WebCeph™ (Assemble Circle Co., Ltd., Gyeonggi-do, Republic of Korea). The following variables were included: facial axis (Ba-Na-Pt-Gn), facial depth (Po-Or/Na-Pg), mandibular plane (Po-Or/Go-Gn), lower facial height (Xi-ANS/Xi-Pm), and mandibular angle (Xi-Pm/Xi-ENA). The measurement definitions are presented in Table 1. The values were transferred to an MS Excel spreadsheet (Microsoft Corp., Redmond, WA, USA) for VERT index calculations. Three groups were established based on the VERT index: brachyfacial (VERT > +0.5), mesofacial (VERT ≈ 0), and dolichofacial (VERT < − 0.5). We defined the articular eminence angle as described in a previous study [21]. Additionally, skeletal classes were defined by the sagittal relationship between the maxilla and mandible, assessed using the ANB angle. Class I represents a normal relationship (ANB between 0° and 4°), Class II a retrusive mandible relative to the maxilla (ANB > 4.0°), and Class III a protrusive mandible in relation to the maxilla (ANB < 0°).

**Table 1:** Description of cephalometric measurement definitions.

Linear measurements	Definition
Facial axis	Angle formed between the line from basion to nasion and the line from pterygoid to gnathion
Facial depth	Angle formed between the porion–orbitale plane and the line
Mandibular plane	Angle formed by the porion–orbitale plane and the line from gonion to gnathion
lower facial height	Angle formed between the line from Xi to the anterior nasal spine (ANS) and the line from Xi to suprapogonion
Mandibular arch	Angle formed by the lines from the center of the condyle (DC) to Xi and from Xi to suprapogonion

Measurement reliability

Several studies have shown that CBCT provides precise linear measurements and reliable three-dimensional joint-space assessment, consistent with the accuracy reported by Hajeer et al. [3,22].

In our study, the rater was calibrated by a more experienced clinician, and intra-operator reliability was evaluated through two measurement sessions conducted one month apart. Intraobserver error analysis showed Dahlberg values between 0.73 and 1.15 mm, confirming adequate reproducibility (Table 2). The smallest error was found in the right posterior space (0.73 mm) and the largest in the left anterior space (1.15 mm). All measurements were repeated by the same examiner after an interval of one and a half months using Dahlberg’s formula [23].

**Table 2:** Intraobserver error analysis using Dahlberg’s statistic for condylar space measurements. Values are expressed in millimeters, and lower Dahlberg values indicate greater measurement precision.

First versus second measures	Dahlberg statistics		
	Right	Left	Total
Posterior space (mm)	0.7344	0.7943	0.7649
Superior space (mm)	0.7388	0.8291	0.7853
Anterior space (mm)	0.9195	1.1544	1.0436

Statistical analysis

All analyses were performed using Stata version 15 (StataCorp, College Station, Texas, USA). Descriptive statistics included the calculation of means and standard deviations for the anterior (AS), superior (SS), and posterior (PS) spaces on both sides, as well as for age and sex distribution. Bilateral correlations were assessed with Pearson’s coefficients to evaluate the degree of linear association between the right and left measurements of each variable. To compare the condylar spaces across the brachyfacial, mesofacial, and dolichofacial groups, one-way ANOVA was applied, adjusting for age, sex, and skeletal class when the assumptions of normality and homoscedasticity were satisfied; in cases where these assumptions were not met, the Kruskal-Wallis test was used. Post-hoc pairwise comparisons were adjusted using the false discovery rate (FDR) to control for multiple testing errors.

Cluster analysis of AS, SS, and PS was performed using Ward’s hierarchical method, followed by k-means with k = 3. Cluster validity was assessed through silhouette coefficients and gap statistics. To determine whether condylar space patterns were consistent between both temporomandibular joints, contingency tables were constructed by comparing right and left cluster assignments. The degree of agreement was quantified with Cohen’s kappa (κ), a coefficient that measures concordance beyond chance.

Multivariate models were also applied. Multiple linear regression was used to assess the influence of facial biotype, age, sex, and skeletal class on AS, SS, and PS. Binary logistic regression was performed to estimate the odds of specific outcomes: an “ideal” condylar position defined by Ikeda [1] thresholds (±0.3 mm), bilateral assignment to Group 1, bilateral assignment to Group 2, or bilateral assignment to Group 3. For each model, odds ratios (OR), 95% confidence intervals (CI), pseudo-R² values, the area under the ROC curve (AUC), and Hosmer-Lemeshow goodness-of-fit tests were reported. Finally, the robustness of the logistic regression findings was examined in a sensitivity analysis by varying the tolerance threshold for the “ideal” position (±0.2-±0.3 mm). In addition, side-specific analyses were performed using mixed-effects models, where the patient was specified as a random effect to account for within-subject correlations between joints.

## Results

The analyzed sample consisted of 100 (100%) patients, analyzed bilaterally, with a mean age of 29.7 ± 8.4 years; 63 (63%) were female, and 37 (37%) were male. According to facial biotypes, 39 (39%) patients were brachyfacial, 16 (16%) were mesofacial, and 45 (45%) were dolichofacial (Table 3).

**Table 3:** Mean values of articular and sociodemographic characteristics by facial type. mm: millimeters; dg: degrees. Values are presented as mean ± standard deviation (SD). Analysis of variance (ANOVA) revealed significant differences only in the right anterior space (p = 0.011). The ANOVA test requires the F-value.

Biotype	Total (n = 100)		Brachyfacial (n = 39)		Mesofacial (n = 16)		Dolichofacial (n = 45)		F-value	p-value
	Mean	S.D.	Mean	S.D.	Mean	S.D.	Mean	S.D.		
Condylar distance										
Posterior right (mm)	3.1	1.36	3.05	1.03	2.98	1.11	3.19	1.67	0.18	0.838
Posterior left (mm)	3.08	1.29	3.08	1.13	3.19	1.23	3.04	1.46	0.07	0.931
Medium right (mm)	2.93	1.11	3.09	1.06	3.09	1.2	2.73	1.12	1.27	0.284
Medium left (mm)	3.05	1.06	3.16	0.85	3.37	1.22	2.84	1.13	1.87	0.161
Anterior rigth (mm)	2.49	1.14	2.76	1.24	2.88	1.01	2.12	0.99	4.81	0.01
Anterior left (mm)	2.74	1.08	2.97	1.12	2.77	0.82	2.53	1.11	1.73	0.182
Articular eminence angle										
Right (dg)	38.35	9.51	38.37	10.7	39.34	7.13	37.98	9.32	0.25	0.777
Left (dg)	39.46	8.31	40.17	8.87	39.39	7.53	38.86	8.2	0.12	0.887
Horizontal condylar axis (dg)										
Right (dg)	68.97	10.25	69.47	9.51	68.03	8.99	68.86	11.41	0.11	0.893
Left (dg)	69.24	10.5	29.41	8.27	31.56	7.41	29.22	8.94	1.63	0.200
Age (years)	29.67	8.42	29.41	8.28	31.56	7.42	29.22	8.94	0.48	0.619
Sex										
Female n (%)	63	(63.0%)	25	(39.7%)	8	(12.7%)	30	(47.6%)	1.44	0.487
Male n (%)	37	(37.0%)	14	(37.8%)	8	(12.6%)	15	(10.5%)		

On average, the condylar spaces measured approximately 3.0 mm for the posterior and superior spaces and between 2.5 and 2.7 mm in the anterior space (Table 2). Most measurements showed no significant differences between the biotypes. However, in the right anterior space, the dolichofacial group exhibited smaller values (2.12 ± 0.99 mm) than the brachyfacial (2.76 ± 1.24 mm) and mesofacial groups (2.88 ± 1.01 mm) (p = 0.011). This finding suggests that the dolichofacial biotype is associated with reduced anteroposterior condylar space (Table 2).

Intra- and inter-biotype comparison

The analysis of bilateral symmetry showed that, in the overall sample, the right anterior space was, on average, 0.25 mm smaller than the left (p = 0.047). When stratified by biotype, this difference was significant only in the dolichofacial group (-0.47 mm; p = 0.006), whereas no relevant differences were found in the brachyfacial or mesofacial groups (Table 4).

**Table 4:** Right–left differences in condylar spaces and angles according to facial biotype mm: milimeters; dg: degree. Negative values indicate a reduction on the right side compared with the left side. The analysis revealed significant differences only in the anterior space in the dolichofacial patients. t-paired test requires the t-value.

Biotype	Total (n = 100)				Braquifacial (n = 39)				Mesofacial (n = 16)				Dolichofacial (n = 45)			
	Mean diff.	S.D.	t-value	p-value	Mean diff.	S.D.	t-value	p-value	Mean diff.	S.D.	t-value	p-value	Mean diff.	S.D.	t-value	p-value
Condylar distance																
Posterior (mm)	0.02	0.1	0.196	0.884	-0.03	0.99	-0.191	0.849	-0.2	1.02	-0.807	0.432	0.14	1.07	0.898	0.374
Medium (mm)	-0.12	0.09	-1.307	0.194	-0.07	1.06	-0.434	0.666	-0.27	0.89	-1.249	0.231	-0.18	0.83	-0.864	0.392
Anterior (mm)	-0.25	0.12	-2.006	0.047	-0.2	1.62	-0.793	0.432	0.1	0.79	0.5267	0.606	-0.47	0.98	-2.853	0.006
Articular eminence angle (dg	-1.1	7.71	-1.438	0.153	-1.8	8.24	-1.364	0.181	-0.5	7.76	-0.026	0.979	-0.88	7.33	-0.811	0.425
Horizontal condylar axis (dg)	-0.27	10.21	-0.273	0.785	-0.09	8.81	-0.066	0.947	3	12.04	0.999	0.333	-1.61	10.61	-1.016	0.315

By contrast, the posterior and superior spaces and articular and articular eminence angles did not differ significantly. This indicates that symmetrical condylar space distribution primarily affects the anterior dimension, particularly in patients with a dolichofacial biotype (Table 4).

Multivariate linear models

In the multiple linear regression models (Table 5), the dolichofacial biotype was independently associated with a reduction in anterior space (coef. = -0.52; 95% CI: -1.02, -0.02; p = 0.041) even after adjusting for age, sex, and skeletal class (Table 5).

**Table 5:** Multivariate linear models were used to predict the condylar anterior, superior, and posterior spaces Coef.: linear regression coefficients; 95% CI = 95% confidence intervals, and p-values are shown. The dolichofacial biotype was significantly associated with a reduction in the anterior space. Wald test requires the t-values. .

Variable	Model 1: Anterior space model (R2 = 0.0889)					Model 2: Superior space model (R2 = 0.0923)					Model 3: Posterior space model (R2 = 0.0367)				
	Coef.	IC95% Inf		t-value	p-value	Coef.	IC95% Inf		t-value	p-value	Coef.	IC95% Inf		t-value	p-value
		Lower limit	Upper limit				Lower limit	Upper limit				Lower limit	Upper limit		
Biotype															
Mesofacial	Reference category					Reference category					Reference category				
Braquifacial	0.117	-0.38	0.62	0.46	0.645	0.001	-0.63	0.63	0	0.997	0.043	-0.59	0.68	0.13	0.894
Dolichofacial	-0.521	-1.02	-0.02	-2.07	0.041	-0.378	-1.05	0.3	-1.11	0.269	0.11	-0.59	0.81	0.31	0.755
Skeletal class															
Class I	Reference category					Reference category					Reference category				
Class II	0.552	-0.13	1.24	1.6	0.114	0.596	-0.11	1.3	1.67	0.097	0.232	-0.51	0.97	0.62	0.535
Class III	0.432	-0.32	1.18	1.15	0.254	1.007	-0.32	2.34	1.5	0.136	0.648	-1.33	2.63	0.65	0.517
Age	-0.011	-0.03	0.01	-0.98	0.329	0.017	-0.01	0.04	1.47	0.146	0.003	-0.03	0.03	0.19	0.852
Male	0.195	-0.21	0.6	0.95	0.344	0.205	-0.18	0.59	1.05	0.295	0.391	-0.03	0.82	1.83	0.071
Constant	2.363	1.09	3.63	3.69	<0.001	1.804	0.4	3.21	2.55	0.012	2.17	0.96	3.38	3.55	0.001

By contrast, neither the brachyfacial biotype nor skeletal classes II/III showed a significant association with any condylar space. Males showed a trend toward larger posterior space values (coef. = 0.39; 95% CI: -0.03, 0.82; p = 0.071), although the difference was not statistically significant. Age was not associated with any meaningful variations in the articular space (Table 5).

Identification of condylar patterns

**Figure 2:**
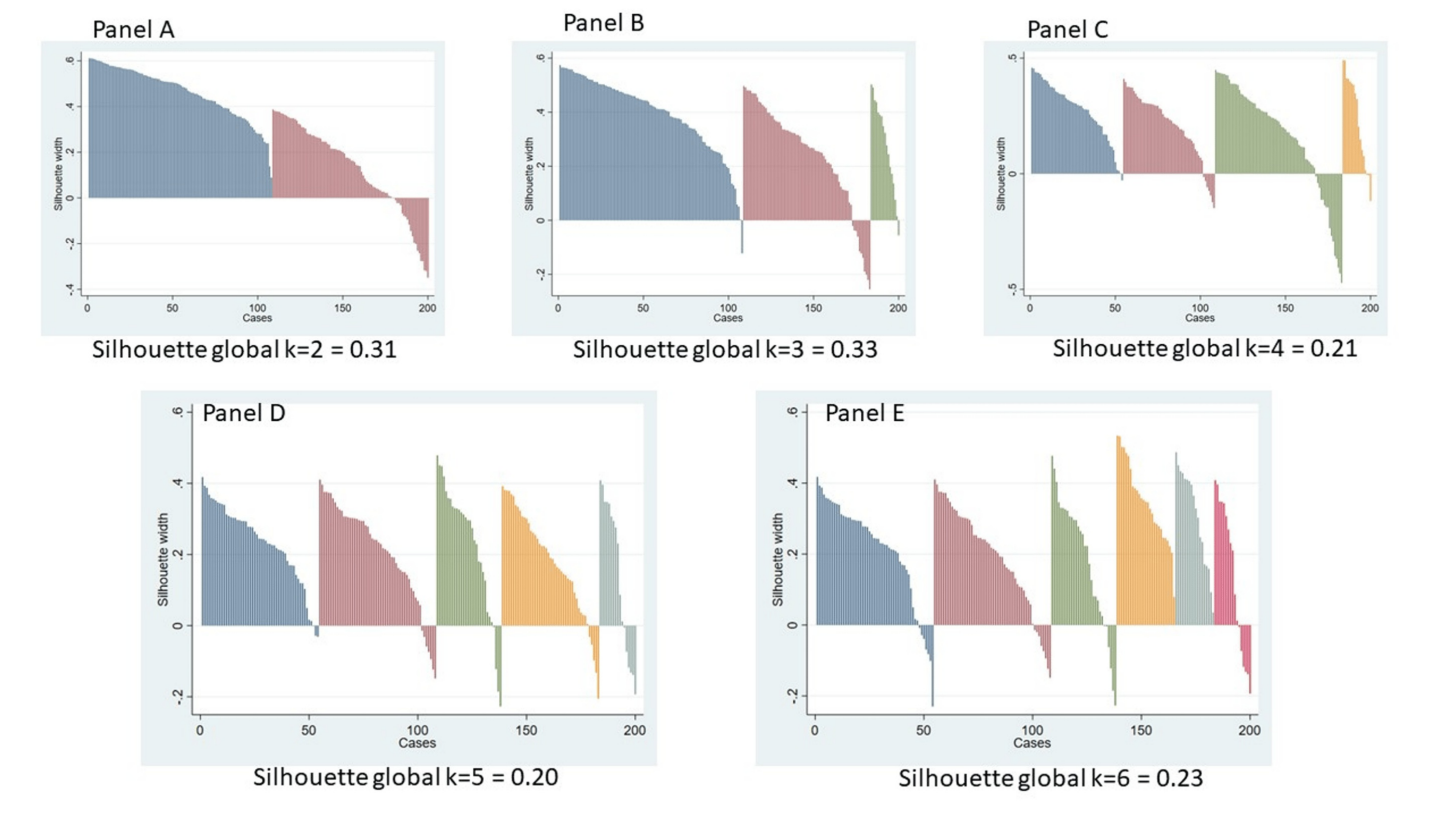
Silhouette plots for hierarchical clustering solutions (k = 2-6) Panel A shows the two-cluster solution, Panel B the three-cluster solution, Panel C the four-cluster solution, Panel D the five-cluster solution and Panel E the six-cluster solution. Each panel displays the silhouette coefficients sorted by case, where positive values reflect good within-cluster cohesion and adequate separation between groups, while negative values indicate unstable or poorly assigned observations. The three-cluster solution (Panel B) presents the highest global silhouette value and fewer negative coefficients, suggesting a more consistent structure and a more reliable classification compared with the k = 2 and k ≥ 4 solutions. Figure 2 is composed of several panels. All graphs and prior analyses were generated using Stata version 15.0 and exported in .jpg format.

The clustering analysis was performed using hierarchical agglomerative clustering with Ward’s linkage and Euclidean distance as the dissimilarity metric. Cluster validity was evaluated using both the silhouette coefficient and the gap statistic across solutions ranging from k = 2 to k = 6. The final cluster structure was selected based on the solution that showed the highest silhouette value, supported by the gap statistic. Silhouette values were 0.31 for k = 2, 0.33 for k = 3, and substantially lower for k≥4 (0.20-0.23). Although k = 2 performed reasonably well, one cluster exhibited weak cohesion (mean ≈ 0.13). The k = 3 solution showed the highest silhouette value and a more balanced internal structure, making it the most stable and interpretable pattern. All silhouette plots (k = 2-6) are presented in Figure 2.

**Figure 3:**
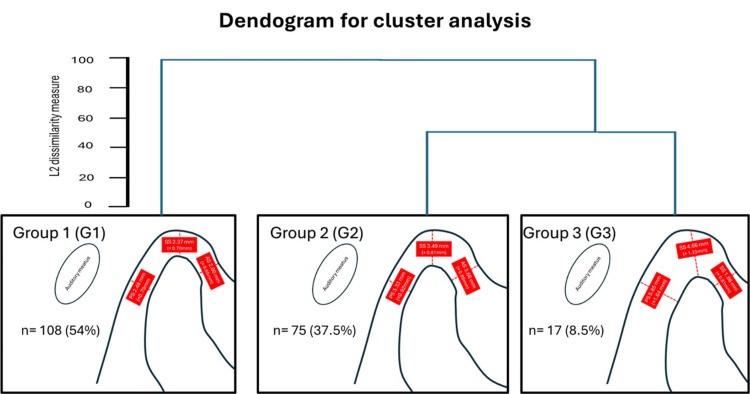
Cluster analysis (k-means) showing three spatial patterns of condylar spaces (AS, SS, and PS). AS: anterior; SS: superior; PS: posterior. Centroids represent the average values for each group. Three patterns were identified: G1 (close to ideal), G2 (moderately enlarged), and G3 (markedly enlarged). Image credits: Dr. Luis Pablo Cruz-Hervert

The cluster analysis revealed three spatial configurations (Figure 3). Group 1 (G1) showed values close to those described by Ikeda, Group 2 (G2) exhibited moderate enlargement of all spaces, and Group 3 (G3) showed marked enlargement, particularly in the superior and posterior spaces.

Bilateral symmetrical condylar space distribution of patterns

**Figure 4:**
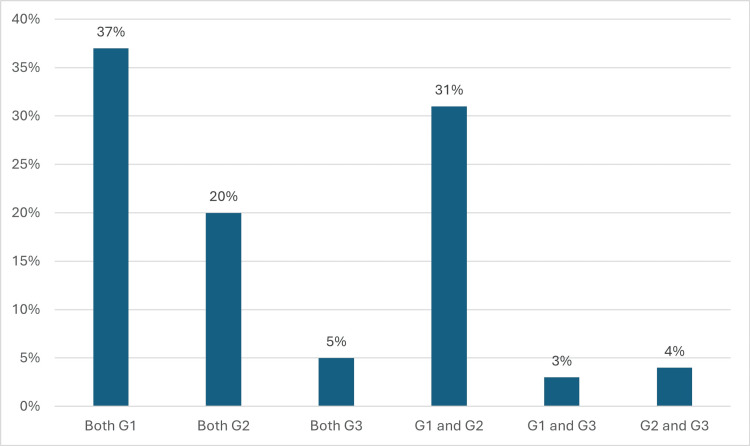
Bilateral distribution of the condylar groups (G1, G2, and G3). Proportions of concordance and combinations between the groups in both joints are shown. Concordance was highest for G1, whereas the G1–G2 combination was the most common. Image Credits: Dr. Luis Pablo Cruz-Hervert

The evaluation of bilateral distribution (Figure 4) showed that 37 (37%) patients presented concordance in G1, 20 (20%) in G2, and five (5%) in G3. The most common combination was G1-G2 (31; 31%), whereas G1-G3 and G2-G3 combinations were less frequent (i.e., 3 (3%) and 4 (4%), respectively). Cohen’s κ coefficient suggested moderate concordance between the joints, indicating that symmetry is not always present within the same patient (Figure 3).

Factors associated with the ideal position and group membership

Multivariate logistic regression models (Table 6) showed that no clinical factor significantly predicted strict compliance with Ikeda’s ideal values (Model A). In contrast, the dolichofacial biotype significantly increased the likelihood of bilateral membership in G1 (OR 4.08, 95% CI 1.06-15.75, p = 0.041) (Model B).

**Table 6:** Multivariate logistic regression models for the prediction of condylar position OR: odds ratios; 95% CI: 95% confidence intervals, NA: not analyzed because it does not show statistical relevance. OR and 95%CI are presented for each predictor. HCA: horizontal condylar axis. Model A: prediction of “ideal” condylar space (Ikeda criteria). Models B–D predicted bilateral membership in cluster groups G1, G2, and G3, respectively. Analyses were adjusted for age, sex, skeletal class, biotype, and articular angle when applicable. The pseudo-R² index was used to estimate the model’s explanatory capacity. Wald test requires the Z-values.

Variable	Model A: Ideal condylar space (pseudo R2= 0.1185)					Model B: Both G1 condylar space (pseudo R2 = 0.1402)					Model C: Both G2 condylar space (pseudo R2 = 0.0447)					Model D: Both G3 condylar space (pseudo R2 = 0.0447)				
	Odds ratio	IC 95% Inf		Z-value	p-value	Odds ratio	IC 95% Inf		Z-value	p-value	Odds ratio	IC 95% Inf		Z-value	*p-value	Odds ratio	IC 95% Inf		Z-value	p-value
		Lower limit	Upper limit				Lower limit	Upper limit				Lower limit	Upper limit				Lower limit	Upper limit		
Biotype																				
Mesofacial	Reference category					Reference category					Reference category					Reference category				
Braquifacial	0.697	0.08	6.39	-0.32	0.749	0.598	0.14	2.59	-0.69	0.492	1.21	0.31	4.77	0.27	0.785	0.421	0.02	8.17	-0.57	0.568
Dolichofacial	1.796	0.33	9.8	0.68	0.499	4.084	1.06	15.75	2.04	0.041	0.378	0.08	1.74	-1.25	0.212	1.012	0.09	11.4	0.01	0.992
Class																				
Class I	Reference category					NA	NA	NA	NA	NA	NA	NA	NA	NA	NA	NA	NA	NA	NA	NA
Clase II	0.394	0.04	3.65	-0.82	0.412	NA	NA	NA	NA	NA	NA	NA	NA	NA	NA	NA	NA	NA	NA	NA
Clase III	2.054	0.1	41.53	0.47	0.639	NA	NA	NA	NA	NA	NA	NA	NA	NA	NA	NA	NA	NA	NA	NA
Age	1.055	0.98	1.14	1.40	0.162	1.023	0.97	1.08	0.81	0.419	1.007	0.95	1.07	0.22	0.823	1.043	0.91	1.20	0.59	0.553
Male	0.141	0.02	1.17	-1.82	0.069	0.375	0.13	1.05	-1.87	0.062	1.127	0.38	3.30	0.22	0.828	1.528	0.12	18.96	0.33	0.741
HCA	NA	NA	NA	NA	NA	0.994	0.94	1.05	-0.20	0.844	1.009	0.95	1.07	0.29	0.77	1.112	1.01	1.23	2.05	0.04
Constant	0.729	0.03	18.48	-0.19	0.848	1.576	0.001	15350.59	0.10	0.923	0.061	0.001	762.68	-0.58	0.562	0.001	0.001	0.001	-2.73	0.006

No consistent predictors were identified for Models C and D (bilateral membership in G2 and G3, respectively). However, articular eminence angle showed a marginal association with bilateral G3 membership (OR = 1.11; 95% CI: 1.00-1.23; p = 0.040).

## Discussion

The position of the mandibular condyle within the glenoid fossa is central to orthodontics and the understanding of craniofacial biomechanics. The AS, SS, and PS spaces have been proposed as objective parameters for assessing the ideal rehabilitation position [1,7]. However, clinical evidence suggests wide inter-individual variability, and the so-called “ideal” values are rarely observed in patients undergoing orthodontic treatment [24,25]. Our study addresses this gap by exploring how facial biotypes influence condylar spaces, whether distribution patterns exist beyond normative values, and the factors that can predict membership in specific configurations.

Reliability of measurements

A critical component of the analysis was the assessment of measurement reliability. The Dahlberg values ranged from 0.73 to 1.15 mm, which aligns with the magnitude of errors previously reported for CBCT-based articular measurements [1,7,26,27]. Although the highest error (~1.15 mm) is relatively large for linear measurements of this scale, no previous studies have systematically evaluated the reliability of the specific Ikeda-based protocol used here, which limits direct comparisons with earlier work. This level of imprecision relates to the effect size observed in anterior-space reduction, and we acknowledge that such variability may attenuate estimated coefficients or introduce minor instability in the cluster solution. Even so, the intraobserver error remained within the acceptable range documented in prior CBCT literature, supporting the technical consistency of the method [3,28,29].

Differences by facial biotype

The most consistent finding was a significant reduction in the anterior space in patients with a dolichofacial pattern, as an independent predictor with limited explanatory power. This result is consistent with the descriptive and multivariate models. Previous studies have reported that dolichofacial individuals tend to present with posterior mandibular rotation and retruded condylar position [30,31], which explains the reduction in AS in our sample.

By contrast, the PS and SS contents did not differ significantly between the biotypes. This suggests that the effect of biotype is not uniform across all three condylar spaces, but is concentrated in the anterior space. Clinically, this may reflect that the vertical facial architecture of patients with a dolichofacial pattern predisposes them to a backward tilt of the mandible, displacing the condyle posteriorly, and narrowing the AS without consistently altering the SS or PS.

The absence of differences in the articular and horizontal condylar axes supports the idea that biotypes exert their influence mainly on spatial relationships rather than on angular parameters. López et al. [32] similarly reported that variability in condylar positioning across biotypes was expressed more in condylar translation than in the morphological changes.

Distribution patterns: evidence of condylar subgroups

Cluster analysis identified three distinct condylar space distribution patterns (G1, G2, and G3), providing a novel perspective compared with studies that focused only on averages. G1 was closely aligned with Ikeda’s reference values, G2 showed moderate enlargement, and G3 exhibited wider spaces, particularly in the SS group.

To the best of our knowledge, only a few studies have applied cluster analysis in this field. Most studies have limited their approach to group means [7,25]. Our findings suggest that the condylar position in orthodontic patients does not follow a single normative pattern but can be differentiated into distinct functional adaptations. This aligns with observations in severe malocclusion cases, in which adaptive condylar trajectories have been described after treatment [29].

Identifying these clinical subgroups opens the possibility of a complementary classification system in orthodontics, focusing more on condylar patterns than on absolute values.

Bilateral symmetry of patterns

Another key finding is that bilateral symmetry is not the norm. Only 37% of patients showed bilateral concordance in G1, whereas the most frequent combination was G1-G2 (31% of patients). Cohen’s κ reflects moderate concordance, confirming that each TMJ should be evaluated individually.

Condylar asymmetry has been well documented in mandibular deformities [8]. However, our study demonstrated bilateral symmetrical condylar space distribution in orthodontic patients without craniofacial asymmetry. This is in line with López et al [32], who reported significant variability between the left and right sides of the condylar position within asymptomatic populations. Clinically, the assumption of bilateral symmetry may lead to an underestimation of relevant findings on one side of the joint. A practical recommendation is to independently assess each condyle during orthodontic diagnosis.

Ikeda’s ideal values: between experimental reference and clinical applicability

One of the clearest results was that only a minority of patients met Ikeda’s “ideal” values (AS ≈ 1.3 mm, SS ≈ 2.5 mm, and PS ≈ 2.1 mm). This finding is consistent with previous reports that highlight the difficulty in applying these values as clinical standards [7,24].

Studies on orthodontic patients and those with Class II, Division 2 malocclusions have shown that the condyle rarely occupies the “ideal” position in otherwise healthy individuals and tends to shift toward a more central position through adaptive remodeling after treatment [24,33]. Thus, the low proportion of “ideal” condyles in our study reflects biological variability in orthodontic populations rather than pathological variability.

This reinforces the interpretation that Ikeda’s values are best regarded as experimental benchmarks for research, with limited direct clinical applicability in heterogeneous patient populations.

Predictive factors for condylar position

Logistic regression models revealed that the dolichofacial biotype was associated with a greater likelihood of bilateral G1 membership (OR = 4.08, p = 0.041). This may appear contradictory because patients with dolichofacial features have reduced AS. However, when all three spaces were considered simultaneously, dolichofacial patients tended to cluster into patterns closer to the reference values. This finding may suggest a compensatory mechanism in which, despite reduced AS, the overall balance of the three spaces is maintained.

Other factors such as age, sex, and skeletal class did not show consistent associations. This finding contrasts with those of studies reporting partial influences of age and sex on condylar morphology [34]. This discrepancy may reflect differences in age range or stricter inclusion criteria of our sample.

The articular angle showed only a marginal association with membership in G3, suggesting that additional anatomical factors may influence extreme configurations of the condylar spaces. However, this association was weak and requires validation in a larger sample.

Clinical and diagnostic relevance

Our findings have several clinical implications: 1) Individualized evaluation: Since bilateral symmetry is not the norm, each TMJ should be assessed separately in orthodontic diagnosis. 2) Biotype interpretation: Dolichofacial patients may require special attention because of their tendency toward reduced AS, potentially predisposing them to joint overload. 3) Patterns over rigid values: identifying condylar subgroups (G1-G3) provides a more realistic diagnostic framework than applying Ikeda’s rigid values. 4) Orthodontic planning: Condylar position should be integrated with facial biotypes in treatment planning, particularly in extreme vertical patterns.

Study limitations

A critical component of the analysis was the assessment of measurement reliability. The Dahlberg values ranged from 0.73 to 1.15 mm, which aligns with the magnitude of errors previously reported for CBCT-based articular measurements [1,7,26,27]. Although the highest error (1.15 mm) is relatively large for linear measurements of this scale, no previous studies have systematically evaluated the reliability of the specific Ikeda-based protocol used here, which limits direct comparisons with earlier work. This level of imprecision relates to the effect size observed in the reduction of the anterior space, and such variability may attenuate estimated coefficients or introduce minor instability in the clustering results. Even so, the intraobserver error remained within the acceptable range documented in prior CBCT literature, supporting the technical consistency of the method.

All CBCT scans were obtained in maximum habitual intercuspation and under a strict protocol for head posture and positioning, which reduces orientation variability but does not eliminate it [35]. Head orientation itself can influence the precision of joint-space measurements, and although all CBCTs were taken with closed-mouth positioning and standardized alignment, the original Ikeda protocol does not include steps for 3D reorientation or condylar adjustment before measurement. Implementing these procedures could further improve reliability and reduce residual error.

AI-based landmark detection may also introduce systematic and random errors, since automated systems such as WebCeph can vary in landmark placement even when intraobserver reliability is adequate. Relevant studies were incorporated to contextualize this potential source of variability [36,37].

Because the CBCT scans were obtained from patients referred for diagnostic or orthodontic purposes, the sample does not represent the full clinical spectrum of the general population. This introduces spectrum bias and limits the generalizability of the findings.

Finally, due to the cross-sectional design, causality cannot be inferred, and any clinical implications should be viewed as hypothesis-generating. Measurement imprecision may also influence cluster assignments, and for this reason, the three-cluster structure should be interpreted descriptively rather than as a definitive classification. This clarification addresses the reviewer’s concern regarding the interpretative boundaries of the clustering framework.

Future perspectives

Longitudinal studies are needed to assess how condylar patterns evolve following orthodontic treatment, and whether group membership predicts the differential risk of temporomandibular disorders. Validation of cluster analysis in diverse populations would help to establish its diagnostic utility. The development of biotype-specific standards may provide more clinically relevant criteria than universal norms.

## Conclusions

This study suggests that facial biotypes may influence condylar spatial relationships, with dolichofacial patients showing a noticeable reduction in the anterior space. However, because the design is cross-sectional, this association cannot be interpreted as causal and should be considered exploratory.

Condylar position did not follow a single configuration. Instead, it grouped into three distinct patterns identified by the clustering analysis, and true bilateral symmetry was uncommon. The proportion of patients who matched Ikeda’s ideal values was low, reinforcing that these parameters function as experimental references rather than clinical norms.

The dolichofacial biotype behaved as an independent predictor with limited explanatory power, while age, sex, and skeletal class showed no consistent influence. These findings do not establish causation, yet they signal trends that warrant confirmation through longitudinal research.

In clinical orthodontics, these observations support a careful and individualized assessment of each temporomandibular joint. The biotype should be viewed as a possible modulator of the condyle fossa relationship, not a determinant. Diagnostic decisions may benefit from considering spatial distribution patterns rather than relying on fixed numerical thresholds or idealized values.
